# The plasma level of mCRP is linked to cardiovascular disease in antineutrophil cytoplasmic antibody-associated vasculitis

**DOI:** 10.1186/s13075-020-02321-w

**Published:** 2020-10-02

**Authors:** Kai-Li Wu, Qing-Hui Liang, Bin-Tao Huang, Na Ding, Bo-Wei Li, Jian Hao

**Affiliations:** 1 Renal Division, Department of Medicine, The Affiliated Hospital of Inner Mongolia Medical University, Huhehot, 010050 Inner Mongolia China; 2 Inner Mongolia Medical University, Huhehot, 010059 Inner Mongolia China

**Keywords:** mCRP, AAV, Cardiovascular diseases, eGFR

## Abstract

**Background:**

C-reactive protein (CRP) has two natural isomers: C-reactive protein pentamer (pCRP) and C-reactive protein monomer (mCRP). The levels of CRP are significantly elevated in patients with anti-neutrophil cytoplasmic antibody (ANCA)-associated vasculitis (AAV). mCRP not only activates the endothelial cells, platelets, leukocytes, and complements, but also has a proinflammatory structural subtype that can localize and deposit in inflammatory tissues. Thus, it regulates a variety of clinical diseases, such as ischemia/reperfusion (I/R) injury, Alzheimer’s disease, age-related macular degeneration, and cardiovascular disease. We hypothesized that plasma mCRP levels are related to cardiovascular disease in AAV.

**Methods:**

In this cross-sectional study, 37 patients with AAV were assessed. Brain natriuretic peptide (BNP) and mCRP in plasma were assessed by enzyme-linked immunosorbent assay (ELISA). The acute ST-segment elevation myocardial infarction (STEMI) was diagnosed by coronary angiography, and the Gensini score calculated. Echocardiography evaluated the ejection fraction (EF%), left ventricular end-diastolic volume (LVEDV), left ventricular end-systolic volume (LVESV), and left ventricular mass index (LVMI). Estimated glomerular filtration rate (eGFR) was calculated based on serum creatinine, age, and gender.

**Results:**

The plasma level of mCRP in AAV was significantly higher than that in healthy volunteers (*P* < 0.001). Then, mCRP and CRP levels were compared with and without STEMI complications in AAV. The plasma level of mCRP was higher, but that of CRP was lower in STEMI. The plasma level of mCRP was correlated with Birmingham vasculitis activity score (BVAS), eGFR, BNP, EF%, LVEDV, LVESV, LVMI, and STEMI complications’ Gensini score in AAV; however, CRP did not correlate with BNP, EF%, LVEDV, LVESV, LVMI, and Gensini score.

**Conclusions:**

The plasma level of mCRP was related to cardiovascular diseases in AAV patients.

## Introduction

Anti-neutrophil cytoplasmic antibody (ANCA)-associated vasculitis (AAV) is commonly characterized by a small amount of immunoglobulin deposition and segmental vascular wall necrotic inflammation [1]. AAV patients are classified into granulomas with polyangiitis (GPA), eosinophilic granulomas with polyangiitis (EGPA), and microvascular polyangiitis (MPA), according to pathological and clinical characteristics [2]. Target antigens for AAV diseases include protease 3 (PR3) and myeloperoxidase (MPO) [3, 4]. The majority of the Chinese patients with AAV are positive for MPO-ANCA [5–7], which activates and damages glomerular endothelial cells (GEnC) in patients with AAV [7–9].

C-reactive protein (CRP) is an acute-phase protein that serves as an inflammatory response biomarker [10, 11]. During activity and relapse of AAV disease, serum CRP levels increase rapidly, followed by a decrease during remission of the disease [12, 13]. Human CRP in blood circulation consists of C-reactive protein pentamer (pCRP) and tissue-related C-reactive protein monomer (mCRP) [14]. In atherosclerosis-related experiments in mice, CRP bound to the Fc receptor CD32, while mCRP bound to another human Fc receptor subtype CD16 in neutrophils and performed function that was opposite to that of CRP [15]. Thus, FC receptor may be the key that links CRP and mCRP to AAV.

The most common pathogenesis of ST-segment elevation myocardial infarction (STEMI) is atherosclerotic fragile plaque rupture and thrombus-induced cardiac muscle cell necrosis. Also, inflammatory reactions are crucial responses [16, 17]. CRP is involved in acute myocardial infarction. mCRP is obtained from the dissociation process of necrobiosis. Moreover, mCRP explains the function of CRP, while pCRP does not promote inflammation [18]. In vitro tests have shown that mCRP induces the synthesis of neutrophil-derived chemokines, stimulates the expression of monocyte integrin [19], and promotes neutrophil adhesion to vascular endothelial cells [20].

CRP is the classical acute-phase protein named for its ability to precipitate and bind to the pneumococcal C-polysaccharide. Although the blood circulation concentration is low in healthy individuals, the level of CRP increases sharply when tissue damage and inflammation occur [21, 22]. Currently, CRP is used not only as a marker of inflammation but also as a crucial and independent predictor of atherosclerotic thrombosis (including cardiovascular events) [22].

Active AAV patients have high levels of CRP [13, 23]. Also, renal involvement is common in patients with AAV, including renal tubulointerstitial (TI) lesions [24, 25]. mCRP is an acute-phase protein in tissues or cells, wherein it might act as an antigen in acute tubular interstitium (ATIN) [25]. Serum CRP is decomposed into mCRP and induces the generation of free oxygen radicals on the surface of apoptotic and necrotic cells, resulting in inflammatory reactions [26]. It was also found that mCRP is not only related to thrombosis [27] and atherosclerotic plaque rupture but can also be concentrated in localized areas after transient myocardial ischemia and related to acute myocardial infarction (AMI) [28]. Therefore, we hypothesized that plasma mCRP levels are related to cardiovascular disease in AAV.

## Methods

### Collecting plasma samples

This cross-sectional study utilized plasma samples collected from 37 patients with AAV and 20 healthy volunteers. The AAV patients were diagnosed in the Department of Nephrology, Affiliated Hospital of Inner Mongolia Medical University, from October 2018 to November 2019. The patients met the criteria set at the Chapel Hill Consensus Meeting [29]. All the participants provided informed consent. Subjects with other autoimmune diseases, such as IgA nephropathy, rheumatoid arthritis, Graves’ disease, and lupus nephritis, as well as secondary and various acute infectious vasculitis, were excluded. A volume of 5 mL venous blood from each patient was withdrawn and collected in EDTA anticoagulation tubes within 24 h of admission. The supernatant was separated by centrifugation at 3000 rpm, 10 min, and stored at − 80 °C for subsequent use. Repeated freeze-thaw cycles are prohibited for this sample. The disease activity was based on the Birmingham vasculitis activity score (BVAS) in AAV patients [30]. We collected baseline patient data including status, age, gender, tobacco, alcohol, hypertension, diabetes, obesity, and biochemical indexes. These cardiovascular risk factors included triglyceride (TG), total cholesterol (Tch), low-density lipoprotein (LDL-C), high-density lipoprotein (HDL-C), CRP, and fibrinogen (Fib). The study was approved by the Ethics Committee of the Helsinki Declaration of Inner Mongolia Medical University.

### Detection of mCRP by enzyme-linked immunosorbent assay (ELISA)

Plasma mCRP levels were tested by ELISA using a commercial kit (Kamaishu Biotechnology Co, Shanghai, China). The 96-well plate was coated with mCRP and blocked to reduce non-specific binding. Antibodies compete for binding to a plate or sample. This kit uses the double-antibody sandwich method to determine the level of human mCRP in the specimen. The microtiter plates were coated with purified human mCRP antibodies to make solid-phase antibodies. Then, mCRP was added to the microwells, followed by the addition of HRP-labeled mCRP antibodies to form an antibody-antigen complex, as assessed by ELISA. Subsequently, the substrate tetramethylbenzidine (TMB) was used to develop the color. The absorbance (OD value) of the colored complex was measured at 450 nm, and the human mCRP concentration in the sample was calculated by a standard curve [31].

### Detection of brain natriuretic peptide (BNP) by ELISA

BNP was detected by a double-antibody sandwich immunoenzyme method, using Unicel-TM-DXI800 (Beckman, CA, USA) full-automated immunoassay instrument. The ELISA was performed, as described previously [32].

### Detection of circulating blood serum creatinine (Scr), estimated glomerular filtration rate (eGFR), and CRP

Scr was determined by a rating method using a commercial kit (Beckman, CA, USA) [33]. The level of CRP was determined by immunoturbidimetry using a commercially available kit (Goldsite, Shenzhen, China) [34]. According to Scr, age, and gender, the eGFR of the patient was calculated. The modification of diet in renal disease (MDRD) formula was adapted by Chinese experts. The female eGFR calculation formula was as follows: eGFR (mL/min/1.73 m^2^) = 186 × Scr − 1.154 × (age) − 0.203 × 0.742 × 1.227 and male eGFR calculation formula eGFR (mL/min/1.73 m^2^) = 186 × Scr − 1.154 × (age) − 0.203 × 1.227 [35].

### Detection of ejection fraction (EF%), left ventricular end-diastolic volume (LVEDV), left ventricular end-systolic volume (LVESV), and left ventricular mass index (LVMI)

The values of EF%, LVEDV, LVESV, left ventricular septal end-diastolic thickness (IVSTD), and left ventricular posterior wall diastolic thickness (LVPWTD) were obtained by echocardiography. Then, the value of left ventricular myocardial mass (LVM) was calculated based on LVIDD, IVSTD, and LVPWTD. Body surface area (BSA) was calculated through weight (*W*) and height (*H*).

LVM (g) = 1.04 {[(LVIDD + PWTD + IVSTD)^3^ − (LVIDD)^3^] − 13.6}.

BSA (m^2^) = (*W*^0.425^ × *H*^0.725^) × 0.007184 [36].

### Statistical analysis

All data were statistically analyzed using SPSS19.0. All grouped data were tested for normality before further analysis. The two groups of enumeration data that conformed to the normal distribution were compared using an independent *t* test. If any of the groups did not match, a non-parametric rank-sum test was used. If the data fulfilled the normal distribution, it would be expressed as means (^*x̅*^) ± standard deviation (SD). The Shapiro-Wilk test, median, and interquartile range (IQR) were used to check the degree of dispersion of the data. The correlation between the two groups of data was analyzed; if both groups met the normal distribution, Pearson’s rank correlation was used, and if any group did not meet the normal distribution, Spearman’s rank correlation was utilized. Cross-tabulation *χ*^2^ test was used for gender comparison. *P* < 0.05 indicated statistical significance.

## Results

### General data from AAV patients and healthy volunteers (normal control)

All the 37 AAV patients, including 17 (45.9%) females and 20 (54.1%) males, harbored MPO-ANCA. Among the 4 AAV patients with STEMI complications, 2 (50.0%) were males and 2 (50.0%) were females, with average age 59.5 ± 14.3 years, while that of the 37 AAV patients was 58.5 ± 10.6 years. The healthy volunteers consisted of 10 (50.0%) males and 10 (50.0%) females, aged 55.3 ± 10.7 years. The comparison of age and sex between 37 AAV patient groups and healthy volunteers (*n* = 20, *P* = 0.266; *n* = 20, *P* = 0.788) did not show significant differences. Next, we used the same method to compare the age and gender of 4 patients with STEMI complications and 33 patients without STEMI complications in the AAV group. Therefore, both the AAV patient group and the normal control, as well as patients with and without STEMI complications in the AAV group, showed no significant difference in the age and sex (*P* > 0.05). Also, the relevant biochemical indicators were analyzed in AAV patients (Table 1).

**Table 1:**
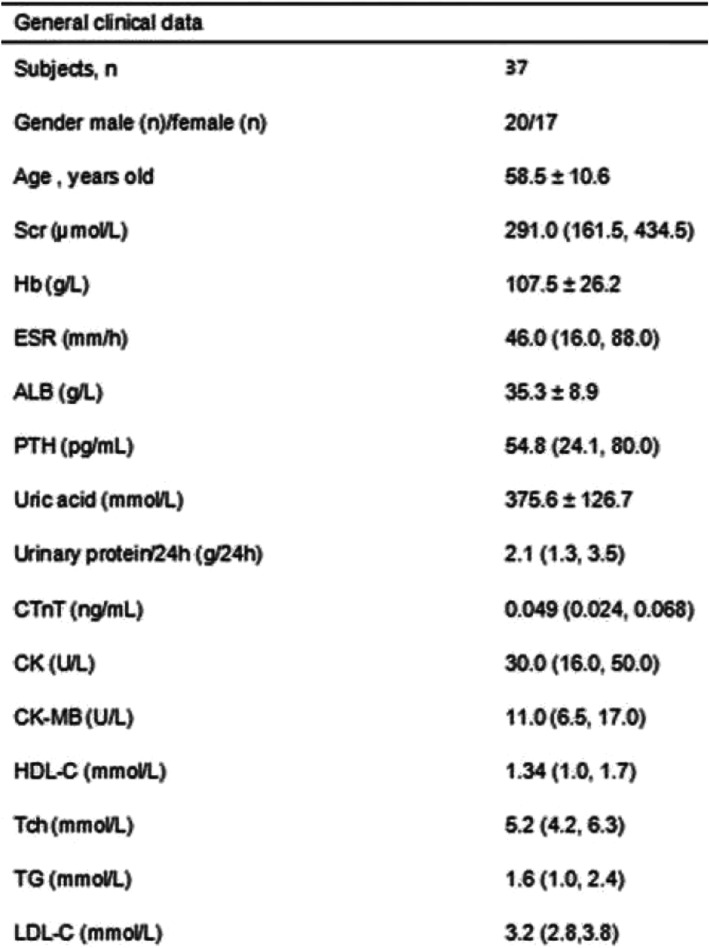
General data (biochemical indexes) in AAV

*Abbreviations*: *Scr* serum creatinine, *Hb* hemoglobin, *PTH* parathyroid hormone, *CTnT* cardiac traponin T, *CK* creatine kinase, *CK-MB* creatine kinase isoenzyme, *HDL-C* high-density lipoprotein, *Tch* total serum cholesterol, *TG* triglyceride, *LDL-C* low-density lipoprotein

### Correlation between mCRP and cardiovascular disease risk factors

The risk factors of cardiovascular diseases include status, age, gender, tobacco, alcohol, hypertension, diabetes, and obesity. Moreover, the correlation between clinical indicators of cardiovascular risk factors (lipid- and inflammation-related factors) and mCRP were compared. We found that mCRP was negatively correlated with total cholesterol (TG) and CRP (*r* = − 0.592, *P* < 0.001; *r* = − 0.340, *P* = 0.040, respectively), (Table 2).

**Table 2:**
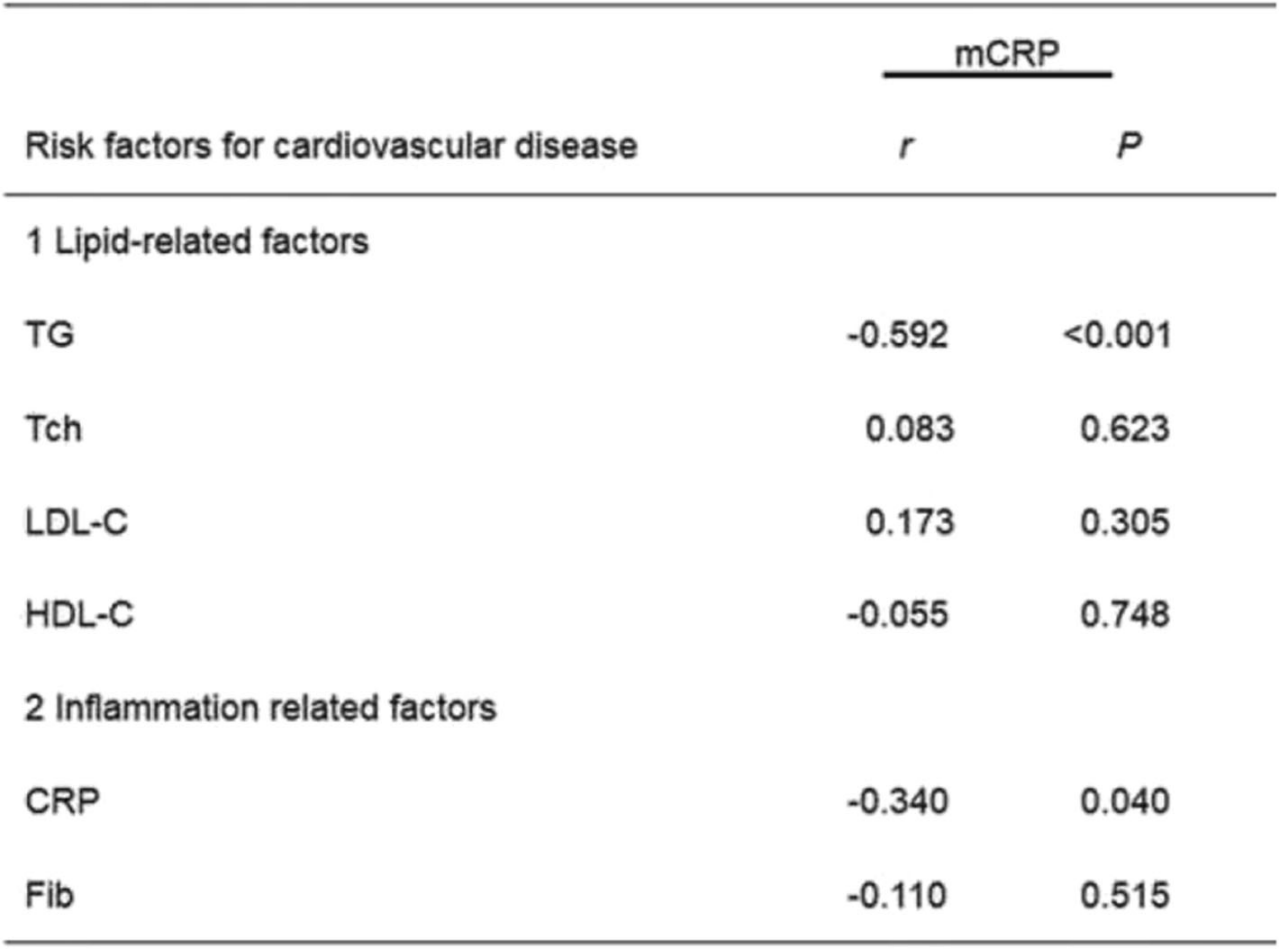
Comparison of the relationship between clinical indicators of cardiovascular risk factors in AAV

*Abbreviations*: *TG* triglyceride, *Tch* total cholesterol, *LDL-C* low-density lipoprotein, *HDL-C* high-density lipoprotein, *CRP* C-reactive protein, *Fib* fibrinogen

### Plasma levels of mCRP were higher in AAV patients than in normal controls

**Fig. 1:**
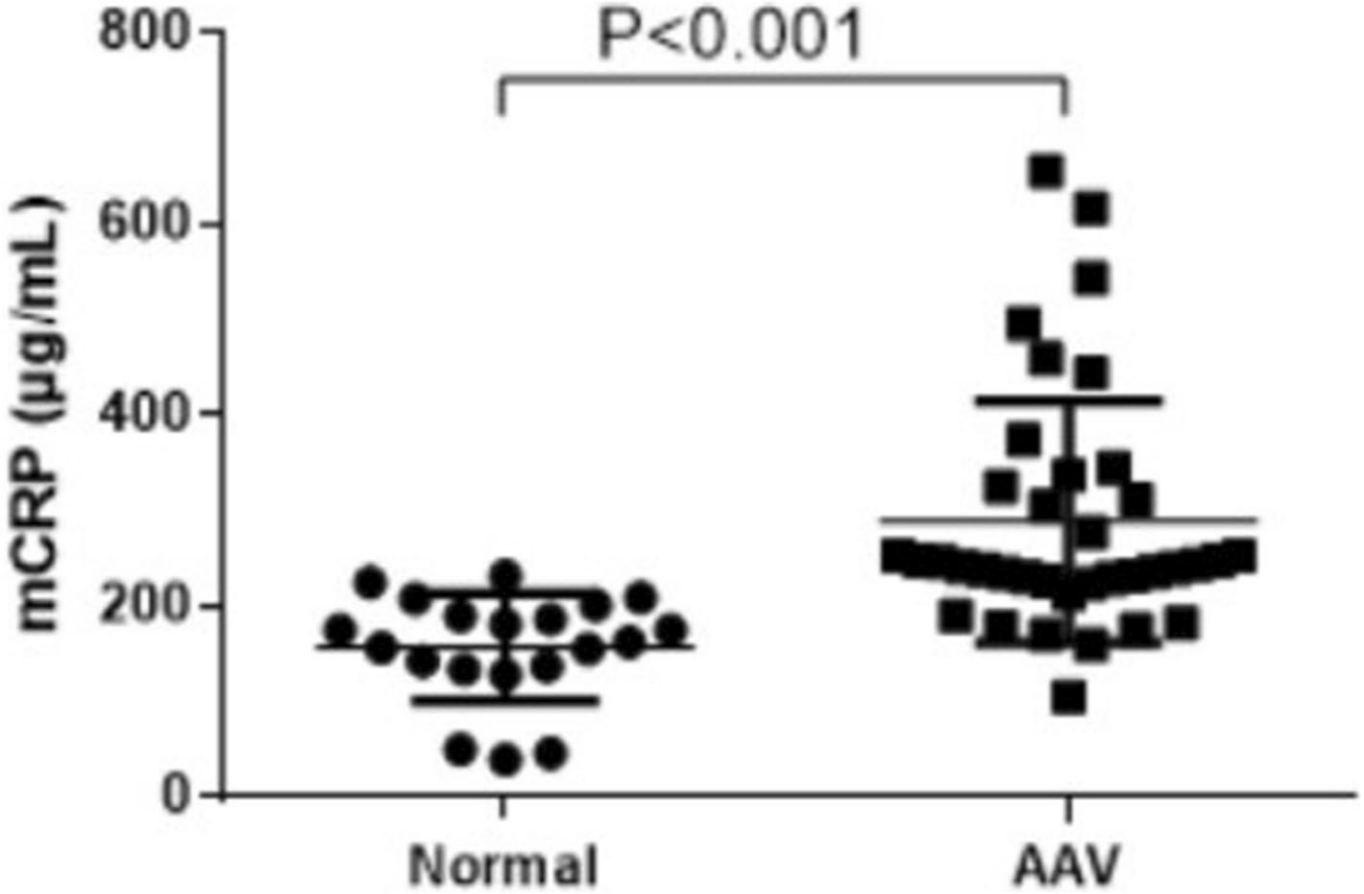
Comparison of the plasma levels of mCRP in AAV patients (*n* = 37) and healthy volunteers (*n* = 20, *P* < 0.001)

The plasma level of mCRP in AAV patients was 244.12 (226.12, 331.725) μg/mL and that in the normal control was 170.0 (135.7, 199.3) μg/mL. Next, we compared the plasma levels of mCRP in AAV patients and healthy volunteers (*P* < 0.001) and found significant differences (Fig. 1).

### Plasma levels of mCRP were highest in patients with than in those without STEMI complications

**Fig. 2:**
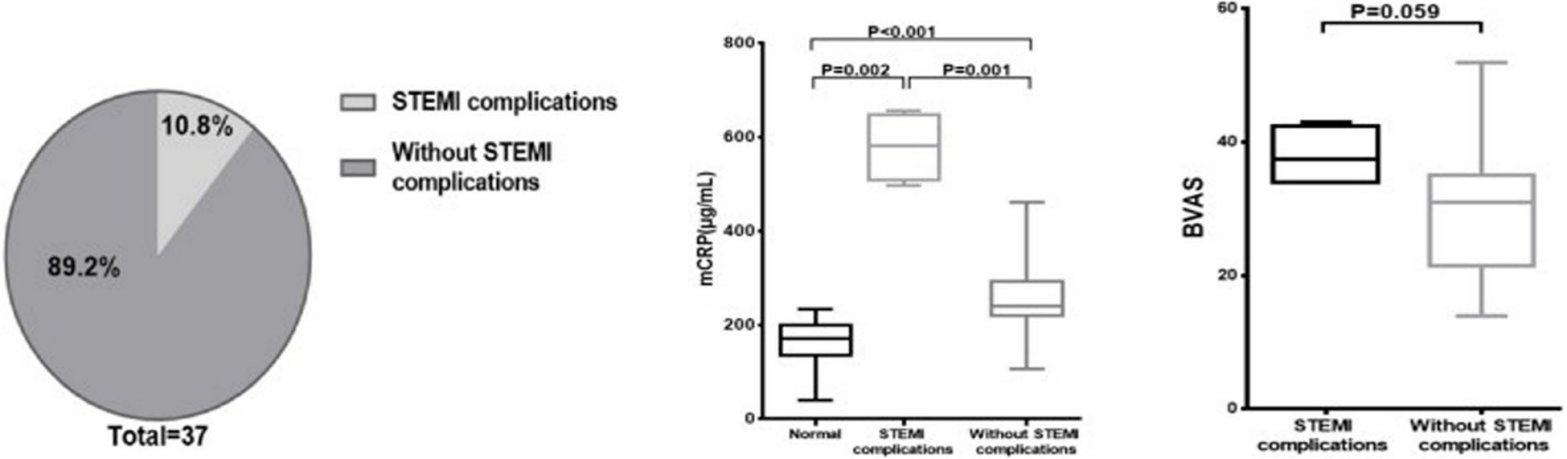
Plasma levels of mCRP were highest in STEMI complications in AAV than without STEMI complications in AAV and normal controls. **a** Thirty-seven AAV patients were divided into two groups: with STEMI complications (*n* = 4) and without STEMI complications (*n* = 33). **b** Plasma levels of mCRP in STEMI complications were highest in the three groups (with STEMI complications in AAV, without STEMI complications, normal). **c** Comparison of BVAS in groups with and without STEMI complications

The AAV patients were divided into groups with (*n* = 4) and without STEMI complications (*n* = 33) groups (Fig. 2a). The plasma concentration of mCRP in patients with and without STEMI complications in AAV group was 581.4 (508.7, 647.3) and 240.8 (219.2, 292.1) μg/mL, respectively, while that in normal controls was 170.0 (135.7, 199.3) μg/mL (Fig. 2b). We also compared the BVAS of patients with and without STEMI complications (Fig. 2c).

### Plasma levels of mCRP were positively correlated with BVAS and Scr and negatively correlated with eGFR

**Fig. 3:**
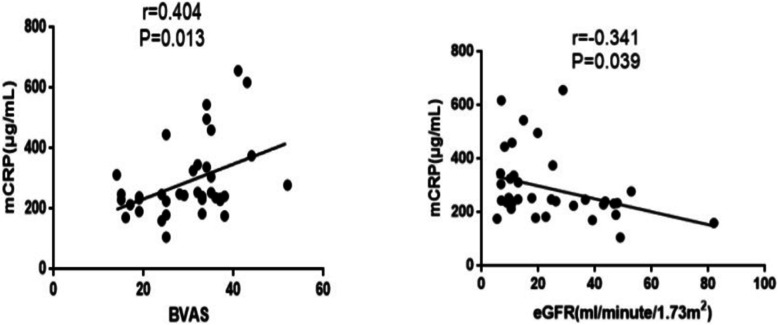
Plasma levels of mCRP were compared with BVAS and eGFR in AAV. **a** Correlation between mCRP and BVAS. The plasma levels of mCRP were positively correlated with BVAS (*r* = 0.404, *P* = 0.013). BVAS, Birmingham vasculitis activity score. **b** Correlation between mCRP and eGFR. The plasma levels of mCRP were negatively correlated with eGFR (*r* = − 0.341, *P* = 0.039). eGFR, estimated glomerular filtration rate

BVAS in 37 AAV patients and normal controls was 29.9 ± 9.1 and 0, while Scr was 291.0 (161.5, 434.5), respectively. The eGFR was calculated based on gender, age, and Scr levels. The eGFR was 17.7 (10.1, 41.0) mL/min/1.73 m^2^ in AAV patients and 170.0 (135.7, 199.3) mL/min/1.73 m^2^ in normal controls. Thus, we analyzed the correlation between mCRP and BVAS (*r* = 0.404, *P* = 0.013, Fig. 3a) and mCRP and eGFR (*r* = − 0.341, *P* = 0.039, Fig. 3b).

### Plasma levels of mCRP were correlated with BNP, EF%, LVEDV, LVESV, LVMI, and STEMI complications’ Gensini score, but not CRP

The Gensini score of patients with STEMI complications in AAV was 72.0 ± 15.1. The BNP level in circulating blood was 155.7 (104.4, 204.3) pg/mL, while the EF (%) levels were 63.0 ± 8.8%, LVEDV was 124.2 ± 25.3 mL, LVESV was 44.2 ± 10.7 mL, LVMI was 86.5 (50.6, 112.6), and the Gensini score of four STEMI complications was 72.0 ± 15.1 in AAV patients.

**Fig. 4:**
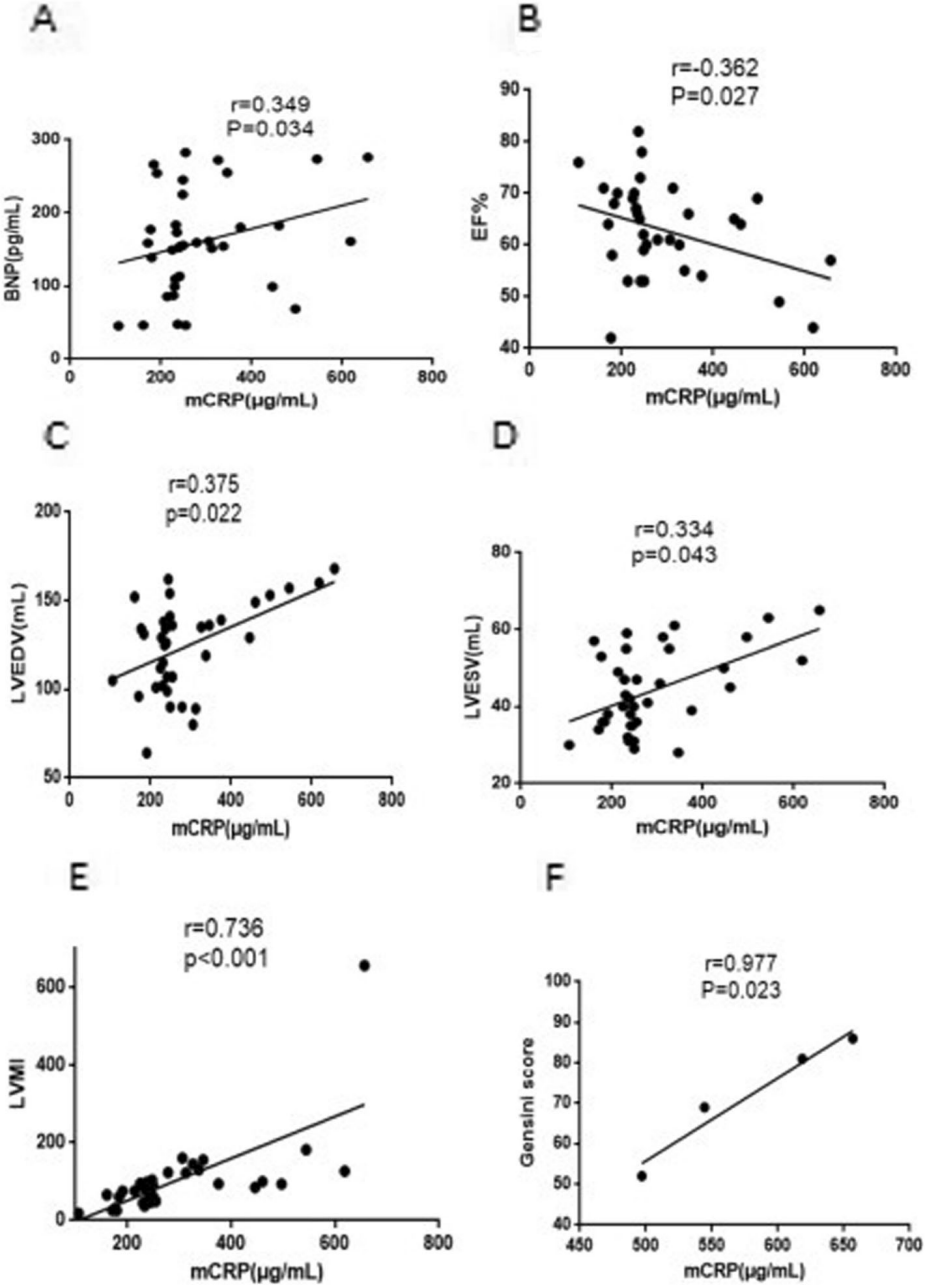
Plasma levels of mCRP were correlated with BNP, EF%, LVEDV, LVESV, LVMI, and Gensini score of STEMI complications in the AAV group. **a** Correlation between mCRP and BNP. The plasma levels of mCRP were positively correlated with BNP (*r* = 0.349, *P* = 0.034). BNP, brain natriuretic peptide. **b** Correlation of mCRP and EF%. The plasma levels of mCRP were negatively correlated with EF% (*r* = − 0.362, *P* = 0.027). EF%, ejection fraction. **c** Correlation between mCRP and LVEDV. The plasma levels of mCRP were positively correlated with LVEVD (*r* = 0.375, *P* = 0.022). LVEDV, left ventricular end-diastolic volume. **d** Correlation between mCRP and LVESV. The plasma levels of mCRP were positively correlated with LVESV (*r* = 0.334, *P* = 0.043). LVESV, left ventricular end-systolic volume. **e** Correlation between mCRP and LVMI. The plasma levels of mCRP were positively correlated with LVMI (*r* = 0.736, *P* < 0.001). LVMI, left ventricular mass index. **f** Correlation between mCRP and Gensini score. The plasma levels of mCRP were positively correlated with Gensini score of STEMI complications in the AAV group (*r* = 0.977, *P* = 0.02). STEMI, ST-segment elevation myocardial infarction

Furthermore, we speculated that the plasma levels of mCRP might be related to the patient’s heart disease, and hence, the correlation between mCRP and BNP, EF%, LVEDV, LVESV, LVMI, and Gensini score of STEMI complications was analyzed (*r* = 0.349, *P* = 0.034, Fig. 4a; *r* = − 0.362, *P* = 0.027, Fig. 4b; *r* = 0.375, *P* = 0.022, Fig. 4c, *r* = 0.334, *P* = 0.043, Fig. 4d; *r* = 0.736, *P* < 0.001, Fig. 4e; *r* = 0.997, *P* = 0.023, Fig. 4f, respectively), while CRP was not correlated (Table 3).

**Table 3:**
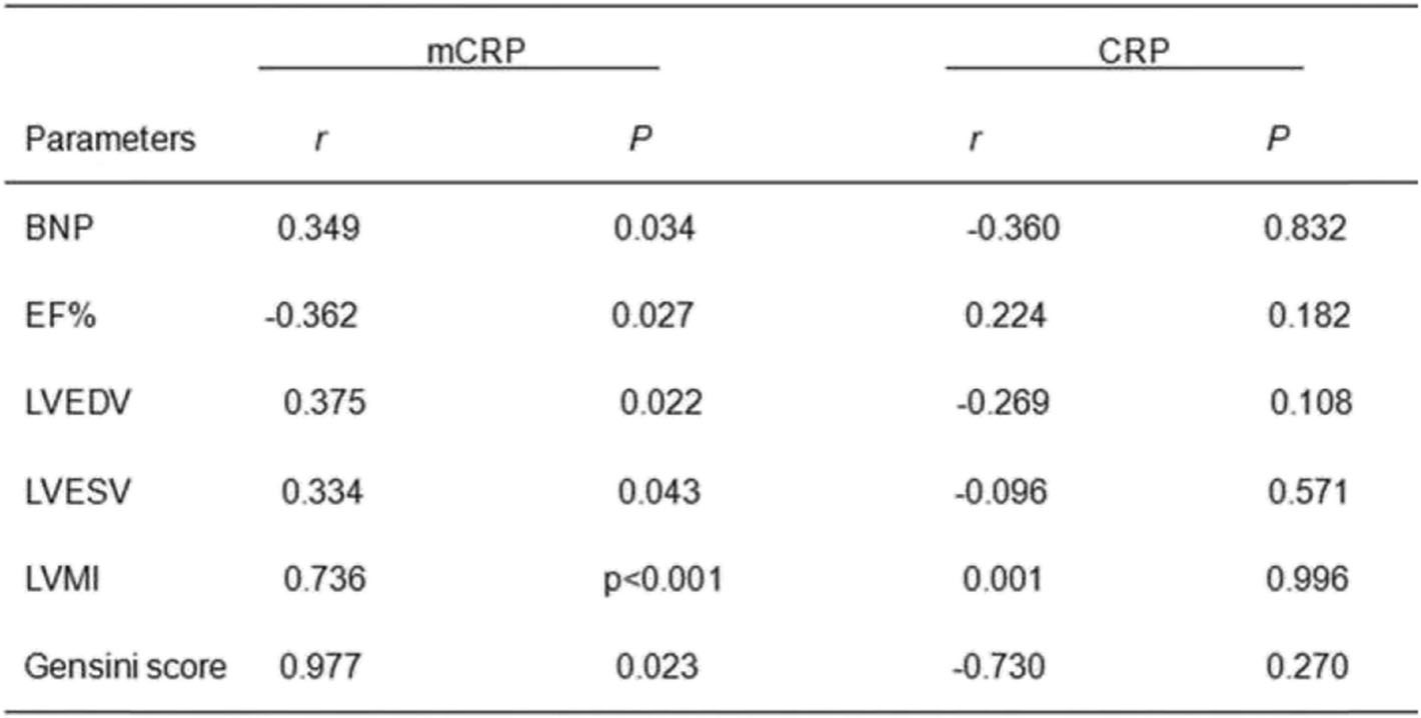
Comparison of mCRP and CRP with BNP, EF%, LVEDV, LVESV, LVMI, and Gensini score with STEMI complications

*Abbreviations*: *BNP* brain natriuretic peptide, *EF%* echocardiography: ejection fraction, *LVEDV* left ventricular end-diastolic volume, *LVESV* left ventricular end-systolic volume, *LVMI* left ventricular mass index

## Discussion

Accumulating evidence has shown that CRP is pathogenic in atherosclerosis, acute myocardial infarction, cerebral infarction, and AAV disease [37]. CRP is one of the biomarkers for assessing AAV disease activity. The serum CRP concentration increased during the AAV active phase and decreased rapidly with the remission of the disease [13, 38]. mCRP can bind with complement factor H (CFH) and is more effective than pCRP in inhibiting alternative complement [39] and plays a critical role in the development of AAV [40, 41]. Xu et al. showed that MPO inhibits the binding between CFH and mCRP, thereby inhibiting the regulatory activation of alternative complements. PR3 does not bind to either pCRP or mCRP, while MPO can bind to mCRP, which blocks the binding between mCRP and CFH [41]. Therefore, the mechanism of mCRP and AAV disease is indistinguishable.

CRP can be decomposed into mCRP at high temperature, urea, or acidic microenvironment [42, 43]. mCRP activates platelets [20], monocytes [44], and endothelial cells [45]. It is a key substance that promotes inflammatory response [21, 46]. The mechanism underlying the inflammatory response of endothelial cells is caused by the interaction of mCRP with neutrophils, macrophages, and platelets [46, 47]. Furthermore, mCRP binds to CFH and directs it to the damaged part of the cell [48, 49], inactivates C3b, and limits the progression of inflammation [39]. Therefore, we speculated that the role of mCRP in the pathogenesis of AAV disease might be related to CFH.

In patients with lupus, the level of anti-mCRP autoantibodies is related to the degree of renal interstitial lesions [50], and mCRP has been reported as an autoantigen in interstitial nephritis-associated uveitis (TINU) syndrome [51]. In AAV patients, anti-mCRP antibodies may be the cause of severe TI lesions [25].

Serum CRP levels increase due to acute infection, trauma, and inflammation [52]. Thus, CRP is commonly used for the risk stratification of cardiovascular disease [53, 54], as well as a marker of generalized atherosclerosis [54, 55]. CRP levels are significantly increased in cardiovascular patients without any symptoms, obvious cardiovascular disease, unstable angina pectoris, myocardial infarction, and other diseases [22, 56]. Also, it is directly involved in the response process of acute myocardial infarction [18]. Thielle et al. demonstrated that mCRP stimulates inflammation, while pCRP has no proinflammatory effect [45]. In addition, Diehl et al. detected mCRP deposition in human atherosclerotic plaque vessels [57]. Similarly, studies on cardiovascular disease have found that mCRP is present in endothelial cells cultured in vitro and can promote inflammatory responses [20]. In the current study, 37 patients with AAV were collected and 4 patients with STEMI complications were found.

mCRP has lectin-like properties and can bind galactose-containing residues [12, 58, 59]. ANCA-induced NETs activate the platelets and then promote mCRP formation on these platelets. Subsequently, the newly generated mCRP can further enhance thrombosis and inflammatory response during platelet activation. mCRP may be a potential link between thrombosis and inflammation in AAV. Therefore, we suspected that the occurrence of STEMI complications in patients was related to mCRP [12]. AAV exhibits various echocardiographic characteristics, such as decreased systolic function, increased diastolic dysfunction, and pulmonary hypertension at the time of diagnosis [60]. The current study focused on cardiovascular disease in patients with AAV, which reflected the clinical examination of cardiovascular function by echocardiography. EF%, LVEDV, and LVESV are indicators of heart function. LVMI is associated with the mortality of patients with chronic kidney disease (CKD) and is a high-risk factor of heart failure [61]. Therefore, we analyzed the correlation between EF%, LVEDV, LVESV, LVMI, and mCRP. In this study, we proved that circulating blood levels of CRP were significantly higher in AAV patients than in healthy volunteers. Also, mCRP levels, but not CRP levels, were significantly increased in patients with STEMI complications. Next, we analyzed the correlation between mCRP levels and the Gensini score of STEMI complications and that between mCRP levels, BNP, and EF% in patients with AAV and found that the level of mCRP was positively correlated with the Gensini score and BNP and negatively correlated with EF%.

Nevertheless, the present study had some limitations. First, it was not a longitudinal study because while collecting cardiovascular clinical indicators, a large amount of experimental data were collected first, followed by comparison analysis. Finally, it was found that the mCRP level was related to the different parameters mentioned in this study. However, AAV patients have low morbidity but high mortality [12]. Most AAV patients visit our department for treatment due to abnormal renal function. The patients we collected in this study were MPO-ANCA because it is a predominant condition in Chinese AAV patients [14, 17]. Therefore, follow-up studies are imperative.

## Conclusions

The level of mCRP is related not only to AAV disease but also to the occurrence of cardiovascular disease in patients with AAV. Therefore, mCRP can be used as a plasma biomarker of cardiovascular diseases in AAV.

## Data Availability

The datasets used and/or analyzed during the current study are available from the corresponding author on reasonable request.

## Abbreviations

CRP: C-reactive protein
pCRP: C-reactive protein pentamer
mCRP: C-reactive protein monomer
ANCA: Anti-neutrophil cytoplasmic antibody
AAV: Anti-neutrophil cytoplasmic antibody-associated vasculitis
PR3: Protease 3
MPO: Myeloperoxidase
Scr: Serum creatinine
Hb: Hemoglobin
PTH: Parathyroid hormone
CTnT: Cardiac troponin T
CK: Creatine kinase
CK-MB: Creatine kinase isoenzyme
HDL-C: High-density lipoprotein
Tch: Total serum cholesterol
TG: Triglyceride
LDL-C: Low-density lipoprotein
Fib: Fibrinogen
ELISA: Enzyme-linked immunosorbent assay
TMB: The substrate tetramethylbenzidine
BNP: Brain natriuretic peptide
STEMI: ST-segment elevation myocardial infarction
EF%: Ejection fraction
LVEDV: Left ventricular end-diastolic volume
LVESV: Left ventricular end-systolic volume
LVMI: Left ventricular mass index
AMI: Acute myocardial infarction
LVIDD: Left ventricular end-diastolic diameter
IVSTD: Left ventricular septal end-diastolic thickness
LVPWTD: Left ventricular posterior wall diastolic thickness
LVM: Left ventricular myocardial mass
GEnC: Glomerular endothelial cells
TI: Tubulointerstitial
ATIN: Acute tubulointerstitial
eGFR: Glomerular filtration rate
BVAS: Birmingham Vasculitis Activity Score
